# Pragmatic Use of Thyroid Autoantibodies in Clinical Practice: An Update

**DOI:** 10.17925/EE.2026.22.1.3

**Published:** 2026-02-05

**Authors:** Naima Parveen, Sachin Chittawar, Deepak Khandelwal, Sameer Aggarwal

**Affiliations:** 1. Harmony-Dr Sachin’s 360 Degree Diabetes Care Centre, Bhopal, MP, India; 2. Harmony-Dr Sachin’s 360 Degree Diabetes Care Centre, Bhopal, MP, India; 3. Khandelwal Diabetes, Thyroid & Endocrinology Clinic, Paschim Vihar, Delhi, India; 4. Dr Sameer Aggarwal’s Endocrine Centre, Rohtak, Haryana, India

**Keywords:** Autoimmune diseases, Graves’ disease, Hashimoto’s thyroiditis, thyroglobulin, thyroid peroxidase, thyrotropin receptor

## Abstract

Autoimmune thyroid diseases, encompassing Hashimoto’s thyroiditis and Graves’ disease, are driven by immune-mediated targeting of thyroid antigens. Central to their diagnosis and management is the detection of circulating thyroid autoantibodies (TAbs), particularly those against thyroid peroxidase (TPO), thyroglobulin (Tg) and the thyrotropin receptor (TR). These antibodies have traditionally served as disease markers, but emerging evidence underscores their functional roles in pathogenesis, prognostication and therapeutic decision-making. For example, TR antibodies not only confirm the diagnosis of Graves’ disease but also help assess disease activity, predict treatment response and evaluate foetal risk during pregnancy. Similarly, TPO and Tg antibodies contribute to the identification of subclinical thyroid dysfunction and have implications for reproductive health and immune-related adverse events from biologic therapies. Newly identified antibodies targeting molecules, such as pendrin, sodium-iodide symporter and megalin, suggest a broader autoimmune footprint than previously recognized. However, their clinical relevance remains to be fully elucidated. The interpretation of TAb is further complicated by assay variability, dynamic antibody titres and their presence in euthyroid individuals, particularly in special populations such as pregnant women, children and those with other autoimmune disorders. This article synthesizes current insights into the immunological basis, diagnostic performance and clinical applications of TAb. It emphasizes the need for a pragmatic, context-specific approach to testing, advocating for a more tailored integration of antibody data into clinical decision-making. By aligning immunologic knowledge with real-world practice, this article aims to refine the utility of TAb in contemporary endocrine care.

## Highlights

- Autoantibodies refine autoimmune thyroid disorder diagnosis and risk.
- Thyroid-stimulating hormone receptor antibody predicts relapse, severity and foetal impact.
- Thyroid peroxidase antibody and thyroglobulin antibody linked to fertility outcomes.
- Novel antibodies expand thyroid autoimmunity scope.

The interplay between immunology and endocrinology is vividly exemplified in autoimmune thyroid disorders (AITDs), where immune dysregulation targets the thyroid gland through both cellular and humoral mechanisms. Hashimoto’s thyroiditis (HT) and Graves’ disease (GD) represent the predominant phenotypes of these conditions, marked by distinct patterns of thyroid dysfunction: hypothyroidism and hyperthyroidism, respectively.^1–3^ A central and defining feature of AITD is the generation of thyroid autoantibodies (TAbs), which are circulating autoantibodies against key thyroid antigens, most notably thyroid peroxidase (TPO), thyroglobulin (Tg) and the thyrotropin receptor (TR). While these autoantibodies have long served as serological markers for diagnosis, mounting evidence supports their active role in disease pathogenesis, prognosis and treatment stratification.^4,5^

Over recent decades, advances in molecular immunology have expanded the understanding of these autoantibodies beyond their diagnostic value. For example, TR antibodies not only confirm the diagnosis of GD but also predict the risk of relapse after treatment, influence ophthalmopathy severity and guide decisions in pregnancy.^6,7^ Similarly, TPO antibodies (TPOAbs) and Tg antibodies (TgAbs) have implications extending beyond the thyroid axis, including associations with reproductive failure, postpartum thyroiditis and responses to immunomodulatory therapies.^8,9^ The discovery of antibodies targeting lesser known antigens, such as pendrin, sodium-iodide symporter (NIS) and megalin, adds further depth to the evolving landscape of thyroid autoimmunity, though their clinical utility remains less established.^10,11^

Despite this wealth of information, the clinical use of TAb often lacks consistency and nuance. In some settings, serological testing is overutilized without clear clinical indication, while in others, potentially informative antibody data are underused or misinterpreted. Moreover, the significance of antibody positivity in asymptomatic or euthyroid individuals, such as pregnant women, children and patients with other autoimmune diseases, remains a subject of on-going debate. The heterogeneity of antibody functions, the variability in assay performance and the dynamic nature of antibody levels all contribute to this interpretive complexity.^12,13^

Given these challenges, there is a growing need for a pragmatic, evidence-informed approach to the use of TAb in clinical practice. This article aims to provide an integrated perspective on the biology, utility and limitations of established and emerging TAb. By bridging mechanistic insights with clinical relevance, we seek to assist clinicians in making judicious decisions regarding antibody testing tailored to the patient’s presentation, risk profile and therapeutic context. Such a framework not only enhances diagnostic precision but also supports personalized care in AITD.

## Types of thyroid autoantibodies

### Anti-thyroid peroxidase antibody

TPOAbs are widely recognized as a defining feature of AITD, with a higher prevalence in affected individuals compared with other thyroid antibodies.^5^

TPOAbs specifically target TPO by binding to conformational, discontinuous epitopes within distinct immunodominant regions, referred to as immunodominant region (IDR)-A and IDR-B. These regions comprise multiple contact sites, including amino acid residues 225, 353–363, 377–386, 597–604, 611–618, 620, 624, 627, 630, 646, 707, 713–720 and 766–775.^14,15^

Both healthy individuals and those with AITD may possess TPOAbs that recognize similar antigenic sites; however, a critical distinction lies in their functional impact. In autoimmune conditions, TPOAbs exhibit pathogenic properties; they can activate complement cascades, mediate thyrocyte lysis and inhibit TPO enzymatic activity through competitive binding. In contrast, TPOAb in healthy individuals typically lack these cytotoxic effects.^16^

Furthermore, TPOAbs are implicated in promoting oxidative stress, particularly in HT. Nonetheless, their pathological role in GD remains less clearly defined. Structural studies suggest that effective interaction between TPO and its antibodies may necessitate significant alterations in the antigen’s tertiary structure, influenced by epitope spatial configuration, domain organization and membrane anchoring.^17,18^

TPOAbs are predominantly of the immunoglobulin G (IgG) class, with subclass distribution observed in the following order: IgG1 (70%), IgG4 (66.1%), IgG2 (35.1%) and IgG3 (19.6%). In some cases, immunoglobulin A (IgA)-type TPOAbs have also been detected, albeit at lower concentrations.^19^

### Anti-thyroglobulin antibody

The immunostimulatory capacity of Tg is influenced, in part, by its endogenous thyroid hormone (TH) content, specifically thyroxine (T4) and triiodothyronine (T3). Variations in the concentration of these hormones within the Tg molecule can induce conformational shifts, unveiling or concealing distinct antigenic determinants (epitopes). As a result, the ability of autoantibodies to bind Tg is modulated by the hormonal saturation of the protein.^20^

In individuals without AITD, TgAbs predominantly recognize conserved epitopes, especially those located within T4- and T3-binding regions of Tg. In contrast, TgAbs in patients with AITD exhibit broader specificity, targeting a more diverse set of antigenic sites. These antibodies primarily identify conformational epitopes shaped by the three-dimensional structure of Tg, while linear epitopes are recognized to a lesser extent. This pattern suggests that the fragmentation of Tg enhances its immunogenicity, likely by exposing structurally complex conformational regions.^21,22^

TgAbs are typically polyclonal and mainly belong to the IgG class, with subclass distribution showing a descending order of prevalence: IgG4>IgG3>IgG2>IgG1. Minor proportions of other immunoglobulin types, including IgA and light chains (kappa and lambda), have also been detected in some cases.^23^

The humoral immune response against Tg appears to be focused on two major immunodominant regions. These epitopes are largely confined to the central domain and the C-terminal portion of the protein, particularly encompassing residues 143, 144, 147, 150–154, 153 and 155–159.^24^

Generation of TgAb may be triggered by the abrupt release of Tg into the extracellular environment, often following thyroid tissue injury due to inflammation (such as thyroiditis), mechanical trauma or surgical manipulation. Additionally, the iodine status of the thyroid plays a critical role in Tg immunogenicity. Notably, excessive iodine intake has been shown to alter the structure of Tg, thereby increasing its antigenic potential and possibly enhancing autoimmune reactivity.^25,26^

### Thyroid-stimulating hormone receptor antibodies

Just as TPOAbs are regarded as the hallmark of HT, thyroid-stimulating hormone receptor antibodies (TRAbs) are considered the signature serological marker of GD. While TRAbs are detected in approximately 90–95% of cases with GD, their prevalence in HT is relatively lower, ranging from 10% to 20%. Therefore, TRAb testing plays a critical role in differentiating the aetiology of hyperthyroidism in clinical practice.^27^

The hyperthyroid state observed in GD is primarily driven by TRAb that mimic the physiological action of thyroid-stimulating hormone (TSH) on thyroid follicular cells. These antibodies activate the TR, a seven-transmembrane G protein-coupled receptor expressed predominantly on thyrocytes and also present on thymocytes and orbital fibroblasts.^7,28^

Functionally, TRAbs are classified into three categories: stimulating, blocking and neutral. Among these, stimulating TRAbs are the most prevalent in GD. These bind to the extracellular N-terminal domain of TR, initiating TH synthesis and secretion independent of the regulatory feedback loop of the hypothalamic–pituitary–thyroid axis.^29,30^

Despite their strong affinity for the receptor, TRAbs are typically present in low serum concentrations. This may be due to their production being restricted to a limited population of B lymphocytes and antigen-presenting cells. Interestingly, in some individuals, the immunological profile may evolve over time from a predominance of stimulatory TRAb to an increase in blocking or neutral antibodies leading to notable shifts in the patient’s clinical presentation and thyroid function test results.^31,32^

TRAbs comprise a heterogeneous population of IgG molecules capable of recognizing specific epitopes on TR. Their affinity and specificity can vary not only between individuals but also within the same individual over time. Even minor alterations in these properties may significantly influence their functional capacity to activate or inhibit the receptor.^31,33^ Several assay formats are employed for TRAb detection, including competitive binding assays, bioassays and enzyme-linked immunosorbent assays (ELISAs).^34^

**Table 1:** Comparative features of major thyroid autoantibodies (thyroid peroxidase antibody, thyroglobulin antibody and thyroid-stimulating hormone receptor antibody)

Feature	TPOAb	TgAb	TRAb
**Target antigen**	TPO	Tg	TSHR
**Associated diseases**	HT>GD	HT	GD
**Prevalence in AITD**	>90% in HT; ~75% in GD	60–80% in HT	90–95% in GD
**Pathogenic role**	Complement activation, ADCC, enzyme inhibition	Likely limited; marker of immune activation	Stimulates or blocks TSHR signalling
**Diagnostic utility**	Confirms autoimmune hypothyroidism	Adjunct in TPOAb-negative cases	Confirms autoimmune hyperthyroidism
**Prognostic utility**	Predicts progression to hypothyroidism	Indicates risk of future thyroid dysfunction	Predicts risk of relapse or remission
**Clinical relevance in special situations**	Miscarriage risk, drug-induced thyroiditis, postpartum thyroiditis	Useful in iodine-replete areas and functional thyroid cancer follow-up	Assesses foetal risk in pregnancy, orbitopathy severity

*ADCC = antibody-dependent cellular cytotoxicity; AITD = autoimmune thyroid disease; GD = Graves’ disease; HT = Hashimoto’s thyroiditis; Tg = thyroglobulin; TgAb = thyroglobulin antibody; TPO = thyroid peroxidase; TPOAb = thyroid peroxidase antibody; TRAb = thyroid-stimulating hormone receptor antibody; TSHR = thyroid-stimulating hormone receptor.*

- **Competitive immunoassays** identify all TRAb subtypes by assessing their ability to displace a labelled ligand (TSH or monoclonal antibody) from the receptor.
- **Bioassays** evaluate the functional activity, stimulatory or blocking, of TRAbs by quantifying intracellular cyclic adenosine monophosphate (AMP) levels generated in TR-expressing cells.
- **ELISA-based methods**, such as the M22 inhibition assay, measure TRAb activity based on their ability to prevent the binding of the monoclonal human TRAb (M22) to thyroid-stimulating hormone receptor (TSHR).^28,34,35^

Comparative features of major TAbs (TPOAb, TgAb, TRAb) are shown in *Table 1*.

### Emerging or less common antibodies

#### Anti-sodium-iodide symporter antibodies

The NIS, a transmembrane glycoprotein located on the basolateral membrane of thyroid follicular cells, facilitates active iodide uptake from the circulation – a critical step in TH biosynthesis. Anti-NIS antibodies (NISAbs) are autoantibodies directed against extracellular domains of NIS and have been identified in a subset of patients with AITD, particularly HT and GD.^10,11^

NISAbs are believed to impair iodide uptake either by sterically hindering iodide-binding sites or by promoting internalization of the NIS protein from the cell membrane. *In vitro* studies demonstrate that NISAb-positive sera can reduce iodide transport efficiency, potentially leading to subclinical or overt hypothyroidism despite structurally intact thyroid tissue.^36^

#### Anti-pendrin antibodies

Pendrin (encoded by *SLC26A4*) is an apical iodide/chloride exchanger located on the luminal membrane of thyrocytes, facilitating the transport of iodide into the follicular lumen for organification. Pendrin antibodies (PDNAbs) have been reported in autoimmune thyroiditis and were more recently described in cases of differentiated thyroid carcinoma.^37,38^

PDNAbs are proposed to inhibit pendrin function, thereby disrupting apical iodide efflux and impairing Tg iodination. Pendrin is also expressed in the inner ear and kidneys, which raises the possibility of cross-organ autoimmunity in susceptible individuals.^38,39^

While current understanding of PDNAb is limited, their detection may help explain persistent hypothyroidism in patients with otherwise normal TPOAb and TgAb profiles. Furthermore, their presence may offer insights into the pathophysiology of iodide organification defects and radioiodine uptake abnormalities in some patients with AITD or thyroid carcinoma.^11,40^

#### Anti-megalin antibodies

Megalin, a multiligand endocytic receptor expressed on the apical surface of thyroid follicular cells and proximal renal tubules, plays a key role in the reabsorption of TH-bound proteins and in the internalization of Tg during colloid resorption. Megalin antibodies (MegAbs) have been identified in animal models and a small number of human studies, particularly in autoimmune thyroiditis and systemic lupus erythematosus (SLE).^41^

MegAb may impair Tg uptake, interfere with colloid processing and potentially contribute to both thyroidal immune activation and renal involvement in systemic autoimmunity. In murine models, MegAbs have been shown to deposit in glomeruli, suggesting a pathogenic link between thyroid and kidney autoimmunity.^42^

Although their detection in human thyroid disease remains rare and is not part of standard diagnostic workups, MegAbs are emerging as markers of systemic autoimmune involvement and may be relevant in polyendocrine autoimmune syndromes or cases with thyroid–renal axis dysfunction.^43^ A summary of lesser known TAb and their clinical relevance is shown in *Table 2*.

## Pathophysiological basis of autoantibody production

### Immune mechanisms

The generation of TAb arises from a complex interplay between the innate and adaptive immune systems. A central feature involves the loss of self-tolerance to thyroid-specific antigens, such as TPO, Tg and TR. This breach in tolerance leads to antigen presentation by dendritic cells and macrophages, followed by T cell activation, especially T helper type 1 and T helper type 17 subsets. These helper T cells stimulate autoreactive B lymphocytes to proliferate, undergo class switching and differentiate into plasma cells that secrete pathogenic autoantibodies.^4^

**Table 2:** Summary of emerging thyroid autoantibodies: Clinical relevance and research directions

Antibody	Detection methods	Associated conditions	Clinical use (current)	Research priorities
Anti-NIS	Radioligand assay	HT, GD	Experimental	Standardize assays, study prevalence
Anti-pendrin	Immunoblot, ELISA	HT, thyroid cancer	Limited	Link with iodide organification defects
Anti-megalin	Animal models, few human studies	HT, SLE	Theoretical	Explore renal–thyroid axis role
THAb	Radioimmunoassay interference	AITD, APS-3, RA	Confounds TFTs	Develop better interference detection tools

*AITD = autoimmune thyroid disease; Anti-NIS = anti-sodium-iodide symporter; APS-3 = autoimmune polyendocrine syndrome type 3; ELISA = enzyme-linked immunosorbent assay; GD = Graves’ disease; HT = Hashimoto’s thyroiditis; RA = rheumatoid arthritis; SLE = systemic lupus erythematosus; TFTs = thyroid function tests; THAb = thyroid hormone autoantibody.*

Furthermore, follicular helper T cells contribute to the formation of germinal centres within thyroid tissue, sustaining chronic antibody production.^44^ Regulatory T cell (Treg) dysfunction is frequently observed in patients with AITD, enabling the persistence of autoreactive clones.^45^ Cytokines, such as interleukin (IL)-6, IL-21 and interferon gamma, promote autoantibody synthesis, while B cell-activating factor facilitates B cell survival and autoantibody affinity maturation.^46^

### Genetic and environmental triggers

Genetic predisposition plays a pivotal role in susceptibility to autoantibody generation. Polymorphisms in human leucocyte antigen (HLA) class II genes (e.g. HLA-DR3, DR5) and non-HLA loci, such as cytotoxic T-lymphocyte-associated protein 4 (CTLA-4), protein tyrosine phosphatase non-receptor type 22 (PTPN22), Forkhead box protein P3 (FOXP3) and TSHR, are strongly associated with AITD. These genetic variants may alter antigen-presentation thresholds or regulatory immune pathways, tipping the balance towards autoimmunity.^47^

Environmental factors further modulate this predisposition. Notably:

- Iodine excess increases Tg iodination and structural modification, thereby enhancing its immunogenicity.^48^
- Infections (e.g. *Yersinia enterocolitica*, *Helicobacter pylori*, hepatitis C) can trigger molecular mimicry, wherein pathogen-derived peptides resemble thyroid epitopes.^49^
- Stress, smoking and radiation exposure have been implicated in immune dysregulation.^50^
- Female sex hormones, particularly oestrogens, influence autoantibody prevalence, consistent with the marked female predominance in AITD.^51^

Together, these genetic and environmental inputs create a permissive milieu for epitope spreading, wherein immune responses initially directed at a limited antigen broaden to include multiple thyroid autoantigens.

### Role in thyroid tissue damage and dysfunction

TAbs contribute directly and indirectly to tissue injury and glandular dysfunction:

- TPOAb and TgAb participate in complement activation and antibody-dependent cellular cytotoxicity, leading to progressive follicular cell destruction, particularly in HT.^52,53^
- TRAbs, especially stimulatory ones, induce unregulated TH synthesis and secretion in GD by mimicking TSH action on TSHR.^54^
- Blocking TRAbs can inhibit receptor function and cause hypothyroidism in a subset of patients.^55^
- Less common antibodies, such as NISAbs, PDNAb and MegAb, may disrupt iodide transport, colloid resorption or hormone recycling, resulting in altered hormone synthesis or radioiodine resistance.^11^
- Importantly, autoantibodies may also act as biomarkers of on-going immune activity, preceding clinical manifestations of thyroid dysfunction by months or even years. Their persistence or evolution over time (e.g. from stimulatory to blocking TRAb) may predict clinical outcomes or therapeutic responses.^6^

### Clinical role of thyroid autoantibodies in autoimmune thyroid disorders

The widespread presence of TRAb, TPOAb and TgAb affords clinicians a multifaceted toolset. Detection of these immunoglobulins not only confirms AITD but also aids in:

- **Differential diagnosis** – separating Hashimoto-related thyrotoxicosis from classic Graves' hyperthyroidism when clinical features overlap.
- **Therapeutic planning** – estimating the likelihood of response to antithyroid medication in GD.
- **Relapse prediction** – very high or persistent TRAb titres point to an elevated risk of recurrence in GD.
- **Long-term outlook** – individuals carrying any major antibody have a heightened probability of developing hypo- or hyperthyroidism over time, depending on the antibody pattern.^8^

### Clinical implications of thyroglobulin antibodies

In areas where people get enough iodine, testing for TgAb usually does not add much to the usual checks for AITD. However, in places with low iodine levels or where thyroid nodules are common, TgAb testing can be more helpful.

Studies have observed that, in areas where a universal salt iodization programme has been adopted, there has been a marked rise in the detection of TgAb. This trend points to the possibility that excessive iodine intake, primarily through iodized salt, might trigger or enhance the immune response against Tg. As a result, regions with widespread iodization programmes and evidence of high iodine consumption may benefit from TgAb testing as part of public health strategies to evaluate the burden of thyroid autoimmunity and the likelihood of future thyroid dysfunction in the population.^56,57^

Beyond autoimmune thyroiditis, TgAbs play a pivotal role in differentiated thyroid cancer surveillance. Their presence interferes with Tg quantification, sometimes yielding falsely low or undetectable Tg values despite residual or metastatic disease.^58^ Also, trends in TgAb titres can serve as a surrogate marker of disease activity – rising levels suggesting recurrence and declining titres indicating remission.^58^

### Clinical implications of thyroid peroxidase antibodies

Beyond their established role in autoimmune hypothyroidism, TPOAbs hold broader clinical relevance across several contexts. In HT, the presence of TPOAb confirms autoimmune aetiology in individuals with overt or subclinical hypothyroidism and can also predict future thyroid failure in euthyroid individuals.^59^

In reproductive medicine, the role of TPOAb testing remains debated. While the American Thyroid Association (ATA) supports screening in women with infertility or recurrent pregnancy loss, obstetric societies largely remain cautious owing to limited interventional evidence.^60^ Nonetheless, TPOAb positivity identifies women at higher risk of gestational hypothyroidism, miscarriage and preterm birth, even when TH levels are normal.^61^ This has prompted some experts to recommend initiating levothyroxine therapy during pregnancy if TSH levels exceed 2.5 in the presence of positive TPOAb.^9^ Moreover, TPOAb can cross the placenta and may influence foetal neurodevelopment; several studies have noted lower offspring intelligence quotient (IQ) scores, particularly in iodine-deficient settings.^50,61^

TPOAb testing in early pregnancy also helps predict the risk of postpartum thyroiditis, facilitating early monitoring. Outside of pregnancy, TPOAb positivity predicts the eventual development of hypothyroidism in otherwise healthy ageing women, supporting periodic TSH surveillance.^60,61^

Beyond thyroid-specific disorders, TPOAb may be detected in other autoimmune diseases such as SLE, rheumatoid arthritis and type 1 diabetes (T1D), reflecting their nonspecific association with immune dysregulation rather than overt thyroid dysfunction. Additionally, TPOAb testing has expanded relevance in two modern clinical contexts:

- Prior to initiating immune-checkpoint inhibitor therapy, where baseline positivity indicates increased risk for thyroid dysfunction.
- In critical illness, where TPOAb can help distinguish true hypothyroidism from transient TSH elevation.^9^

Finally, individuals with pre-existing TPOAbs are more susceptible to thyroid dysfunction when exposed to certain drugs such as interferon-α, tyrosine kinase inhibitors, IL-2, amiodarone or lithium, underscoring their importance in pre-therapy risk assessment.^9^

### Clinical implications of thyrotropin receptor antibodies

TR antibodies (TRAbs) represent a heterogeneous group comprising stimulating, blocking and neutral immunoglobulins that interact with the TSH receptor (TR). The stimulating subset, known as thyroid-stimulating immunoglobulin (TSI), is the hallmark of GD and drives hyperthyroidism through TSHR activation.^62^ In contrast, TR-blocking antibodies (TBAbs) inhibit TSHR signalling and are occasionally detected in hypothyroidism phases of autoimmune thyroiditis.

Although imaging modalities such as thyroid ultrasound and scintigraphy aid in evaluating hyperthyroidism, TRAb measurement offers a rapid and reliable biochemical method to differentiate GD from other causes, including hashitoxicosis, painless thyroiditis, factitious thyrotoxicosis or toxic nodular goitre.^63^

In thyroid-associated ophthalmopathy (Graves’ orbitopathy), TRAb titres often parallel the severity and activity of ocular disease and may serve as an early predictor of unfavourable outcomes.^64,65^ High TRAb concentrations before radioiodine therapy are associated with an increased risk of orbitopathy exacerbation, and this should be considered in therapeutic planning.^66^

In amiodarone-induced thyrotoxicosis (AIT), a positive TRAb result supports type 1 AIT (iodine-induced GD) rather than type 2 AIT (destructive thyroiditis). Nevertheless, antibody interpretation should always be integrated with clinical and imaging findings for diagnostic accuracy.^67^

Monitoring TRAb titres during antithyroid drug therapy provides additional prognostic information. A progressive decline in TRAb levels usually indicates remission, while a secondary rise may predict relapse of hyperthyroidism. However, serial monitoring is not routinely required, as clinical response remains the principal determinant of therapy adjustment.^12,68^

During pregnancy, TRAb (or TSI) testing assumes particular importance. Measurement in the first and third trimesters is recommended for women with current or past GD to estimate the risk of foetal and neonatal thyrotoxicosis. At delivery, assessing TRAb in cord blood helps guide early neonatal monitoring and management.^69–71^

The blocking subtype (TBAb), though less common, can occasionally provide diagnostic insight in selected scenarios:

1. In patients with HT who remain euthyroid despite minimal levothyroxine requirements, TBAbs may explain transient hypothyroidism due to receptor blockade.
2. In newborns of mothers with HT, TBAb testing aids in distinguishing antibody-mediated neonatal hypothyroidism from other causes.
3. In patients with HT who exhibit features of thyroid-associated orbitopathy, TBAb testing may clarify autoimmune overlap.
4. In patients with long-term hypothyroidism who unexpectedly transition to hyperthyroidism, changing TRAb dynamics (stimulating versus blocking) may underlie the fluctuation.
5. In patients experiencing alternating hypo- and hyperthyroid states, periodic shifts between TSI and TBAb dominance can explain clinical variability.^72–74^

Overall, TRAb testing remains indispensable for diagnosing and characterizing GD and is useful in selected clinical contexts such as pregnancy, orbitopathy and drug-induced thyroid dysfunction. However, routine or serial measurement outside these indications is not warranted, and results should always be interpreted within the broader clinical and imaging context to avoid overreliance or misinterpretation.

### Clinical implications of pendrin antibodies, sodium-iodide symporter antibodies, megalin antibodies and other thyroid autoantibodies

The clinical application of emerging TAbs, such as PDNAb, NISAb, MegAb and TH autoantibodies (THAb) in AITD, remains largely unestablished. At present, there is no robust evidence demonstrating that these antibodies provide diagnostic, prognostic or therapeutic advantages over the well-validated TPOAb, TgAb and TRAb used in HT and GD. Their inclusion in clinical workups without clear evidence may risk misinterpretation and overextension beyond their research intent.

**Table 3:** Evidence-based use of thyroid autoantibodies in diagnostic and therapeutic pathways^6,9,12,13,20,63,67,69,76,81,84,85,89,93,94^

Clinical scenario	Recommended antibody test(s)	How it guides management	Reference(s)
Subclinical hypothyroidism (TSH 4.5–10 mIU/L)	TPOAb	Positive TPOAb suggests higher risk of progression → consider earlier treatment	Vanderpump;^93^ Pearce et al.^94^
Pregnant woman with TSH >2.5 mIU/L	TPOAb	Positive TPOAb may justify levothyroxine initiation to reduce miscarriage risk	Alexander et al.;^13^ Dhillon-Smith and Coomarasamy^9^
Postpartum monitoring	TPOAb	Positive TPOAb indicates risk for postpartum thyroiditis → schedule follow-up testing	Glinoer;^84^ Stagnaro-Green et al.^85^
Suspected thyrotoxicosis with no palpable goitre	TRAb	TRAb helps distinguish GD from painless thyroiditis	Kahaly et al.^63^
GD in remission	TRAb	Persistently high TRAb = high relapse risk → consider longer medical therapy	Diana et al.;^6^ Struja et al.^12^
Pregnant women with past GD	TRAb	High maternal TRAb → risk of foetal/neonatal thyrotoxicosis → monitor foetal heart rate/growth	Alexander et al.;^13^ Mooij et al.^69^
Type 1 diabetes (euthyroid)	TPOAb, TgAb	Positive antibodies → periodic thyroid monitoring, early detection of AITD	Liu et al.^89^
Abnormal TFTs with normal clinical picture	THAb	Consider THAb interference → repeat TFTs using different assay method	Benvenga et al.;^76^ Ghazal et al.^81^
Thyroid cancer monitoring with Tg-based assays	TgAb	Interferes with Tg measurement → use as a surrogate marker; avoid false reassurance in Tg-negative, Ab-positive cases	Soh and Aw^20^
Patients on interferon, tyrosine kinase inhibitors, amiodarone	TPOAb	Baseline positivity predicts thyroid dysfunction → monitor TSH and symptoms during treatment	Dhillon-Smith and Coomarasamy;^9^ Trohman et al.^67^

*Ab = antibody; AITD = autoimmune thyroid disease; GD = Grave’s disease; TFTs = thyroid function tests; Tg = thyroglobulin; TgAb = thyroglobulin antibody; THAb = thyroid hormone antibody; TPOAb = thyroid peroxidase antibody; TRAb = TSH receptor antibody; TSH = thyroid-stimulating hormone.*

Among these lesser studied entities, THAbs directly target circulating THs (T3 and T4). THAb formation is hypothesized to result from extensive Tg release, which exposes hormonogenic epitopes to immune recognition and triggers a humoral response. Four THAb subtypes have been identified based on the immunoglobulin class and the hormone targeted T4-IgG, T4-immunoglobulin M (IgM), T3-IgG and T3-IgM, with T4-IgG and T3-IgG being the most prevalent.^75,76^

The prevalence of THAb varies significantly across populations. In the general population, their occurrence is estimated to be around 1%, whereas in individuals with AITD, prevalence rises markedly, ranging from 20% to 23% in HT and up to 46% in GD.^77,78^ THAbs have also been detected in patients with other autoimmune conditions, such as Sjögren’s syndrome, rheumatoid arthritis and autoimmune polyglandular syndromes. Their presence in these disorders suggests potential molecular mimicry or cross-reactivity involving shared epitopes, particularly in connective tissues.^79,80^

**Figure 1:**
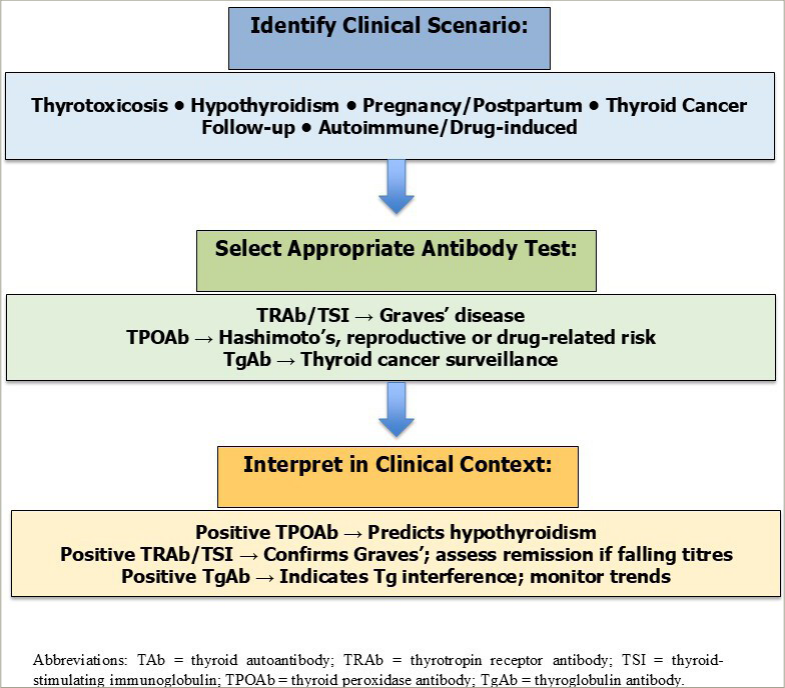
Pragmatic approach to thyroid autoantibody testing in clinical practice

*Tg = thyroglobulin; TgAb = thyroglobulin antibody; TPOAb = thyroid peroxidase antibody; TRAb = thyrotropin receptor antibody; TSI = thyroid-stimulating immunoglobulin.*

THAbs are rarely assessed in routine clinical settings, primarily due to their limited diagnostic relevance and their potential to confound interpretation of thyroid function tests. These antibodies can interfere with peripheral measurements of circulating TH (T3 and T4), leading to misleading results such as spurious elevations or reductions in hormone levels, which do not reflect true thyroid status.^81^

Another antibody identified in patients with AITD targets a second, non-Tg colloidal antigen within the thyroid follicle. However, its functional significance, prevalence and clinical implications remain poorly understood, and further research is needed to elucidate its role in thyroid autoimmunity.^82,83^ In summary, while these antibodies expand the immunological understanding of thyroid autoimmunity, their current role is confined to experimental research. Until standardized assays and prospective validation studies demonstrate reproducible clinical benefit, their use should not be incorporated into diagnostic or therapeutic algorithms, to avoid misinterpretation as clinically validated tools.

## Thyroid autoantibodies in special populations

### Pregnancy and postpartum

Pregnancy represents a unique immunological state that can unmask or modulate AITD. TPOAbs are the most clinically significant in this context and are found in up to 15% of pregnant women.^82^ Their presence is associated with an increased risk of miscarriage, preterm delivery, postpartum thyroiditis and future hypothyroidism.^83^ Routine screening for TPOAbs during the first trimester is advised in women with thyroid dysfunction, infertility or a personal/family history of autoimmunity.^13^ In our view, serial TSH monitoring is recommended throughout pregnancy and the postpartum period in women who are TPOAb-positive, given the increased risk of thyroid failure during gestational and postpartum immune shifts.

TRAb testing is particularly critical in women with a history of GD, regardless of current thyroid status, to assess the risk of foetal or neonatal thyrotoxicosis. High TRAb levels in the second half of pregnancy necessitate close foetal monitoring.^84^

### Paediatrics

In children and adolescents, autoimmune thyroiditis, predominantly HT, is the most frequent cause of acquired hypothyroidism. Detection of TPOAb and/or TgAb supports diagnosis in children presenting with goitre, growth retardation or pubertal delay.^85^ While many children may remain euthyroid initially, antibody positivity predicts progression to hypothyroidism, often warranting annual or biannual thyroid function screening.^86^ The condition may also present in the context of other autoimmune diseases (e.g. T1D, coeliac disease), where routine thyroid antibody screening is advocated as part of comprehensive care.^87^

### Type 1 diabetes and other autoimmune disorders

AITD frequently coexists with other autoimmune conditions, forming autoimmune polyglandular syndromes.^52^ Among individuals with T1D, up to 30% may have positive TPOAb, with a significant proportion developing thyroid dysfunction over time.^88^ Recent evidence from The Environmental Determinants of Diabetes in the Young (TEDDY; Consortium for Identification of Environmental Triggers of Type 1 Diabetes; ClinicalTrials. gov identifier: NCT00279318) study further reinforces this connection, showing that thyroid autoimmunity can appear early in children genetically at risk for T1D, often preceding or co-developing with islet autoimmunity.^89–91^

A summarized algorithm for the pragmatic use of TAb testing in common clinical contexts is presented in *Figure 1*. It provides a step-wise approach linking the clinical scenario to the most appropriate antibody assay.

## Conclusion

TAbs, especially TPOAb, TgAb and TRAb, play a pivotal role in the diagnosis, prognosis and management of AITD. Their utility extends beyond thyroid-specific conditions, offering insights into reproductive health, drug-induced thyroid dysfunction and autoimmune overlap syndromes. While newer antibodies, such as anti-NIS, anti-pendrin and anti-megalin, highlight the expanding landscape of thyroid autoimmunity, their clinical relevance remains limited. Optimal use of antibody testing requires a context-specific approach, particularly in special populations, such as pregnant women, children and those with coexisting autoimmune diseases. Integrating immunological understanding with clinical judgement enhances diagnostic accuracy and supports personalized care in endocrine practice.
